# Intestinal microbiota and biliary system diseases

**DOI:** 10.3389/fcimb.2024.1362933

**Published:** 2024-03-15

**Authors:** Hua Wang, Junfeng Gong, Jingyi Chen, Wei Zhang, Yanjun Sun, Dengqun Sun

**Affiliations:** ^1^ Department of Health, The Chinese People’s Armed Police Forces Anhui Provincial Corps Hospital, Hefei, China; ^2^ Department of General Surgery, The Chinese People’s Armed Police Forces Anhui Provincial Corps Hospital, Hefei, China; ^3^ Department of Pharmacy, The Chinese People’s Armed Police Forces Anhui Provincial Corps Hospital, Hefei, China

**Keywords:** gallstones, primary sclerosing cholangitis, primary biliary cholangitis, biliary tract cancer, microbial community

## Abstract

**Introduction:**

The incidence of biliary system diseases has been continuously increasing in the past decade. Biliary system diseases bring a heavy burden to humanity and society. However, the specific etiology and pathogenesis are still unknown. The biliary system, as a bridge between the liver and intestine, plays an indispensable role in maintaining the physiological metabolism of the body. Therefore, prevention and treatment of biliary diseases are crucial. It is worth noting that the microorganisms participate in the lipid metabolism of the bile duct, especially the largest proportion of intestinal bacteria.

**Methods:**

We systematically reviewed the intestinal microbiota in patients with gallstones (GS), non-calculous biliary inflammatory, and biliary tract cancer (BTC). And searched Pubmed, Embase and Web of science for research studies published up to November 2023.

**Results:**

We found that the abundance of Faecalibacterium genus is decreased in GS, primary sclerosing cholangitis (PSC), primary biliary cholangitis (PBC) and BTC. Veillonella, Lactobacillus, Streptococcus and Enterococcus genus were significantly increased in PSC, PBC and BTC. Interestingly, we found that the relative abundance of Clostridium was generally reduced in GS, PBC and BTC. However, Clostridium was generally increased in PSC.

**Discussion:**

The existing research mostly focuses on exploring the mechanisms of bacteria targeting a single disease. Lacking comparison of multiple diseases and changes in bacteria during the disease process. We hope to provide biomarkers forearly diagnosis of biliary system diseases and provide new directions for the mechanism of intestinal microbiota in biliary diseases.

## 1 Introduction

The biliary is a complete system with independent anatomical structures and physiological functions. It starts from the capillaries between liver cells until the end of the common bile duct opens at the main nipple of the duodenum. The biliary system is the excretory duct of the liver. It is able to transport bile, participate in metabolism, and regulate the internal environment (Schaub et al., 2018). The imbalance of the human immune system and metabolism can lead to the occurrence of biliary diseases, mainly gallstones (GS), biliary inflammation, and biliary tract cancer (BTC) (Maurer et al., 2009; Tazuma et al., 2013). For a long period of time, GS and complications have been extremely common in the biliary. The incidence of GS in domestic and international populations is constantly increasing (Wei et al., 2023). In China, the prevalence of GS has exceeded 12% (Hu et al., 2022). In Western countries, the prevalence of GS among adults is approximately 10%–20% (Morris-Stiff et al., 2023). More than 90% of GS are cholesterol stones and approximately 1:2 between men and women (Xia et al., 2023). There are several types of biliary inflammation, the main ones being cholecystitis and cholangitis. The onset of inflammation is mostly related to stones, which cause obstruction of bile flow, or is accompanied by biliary tract infections. BTC occurs in the canceration of the extrahepatic bile duct and the gallbladder wall. The incidence in men is 1.5 times that in women. Most patients are older than 65 years, and the incidence peaks after 80 years of age. With the development and application of imaging technology, the detection rate of BTC has been increasing year by year (Roa et al., 2022). At present, clinical doctors mostly focus on the symptomatic treatment of biliary tract diseases, but the issue of disease recurrence still needs to be addressed further. The most important challenge is to identify the causes of biliary diseases in order to determine effective prevention and treatment. Early studies have shown that the occurrence and development of biliary diseases are related to genetics and the environment. For example, the adenosine triphosphate binding cassette transporters G5 and G8 (ABCG5/G8) are responsible for the secretion of liver cholesterol, but their variants (*ABCG5-R50* C and *ABCG8-D19 H*) are associated with GS (Lammert et al., 2016). Katsika et al. conducted a correlation analysis of 43,141 pairs of twins with GS and found genetic factors for susceptibility to GS (Katsika et al., 2005). Another study targeting the Indian population found that the *ABCB1* and *ABCB4* germline gene variants are associated with gallbladder cancer, and these variants have also been found in the Chilean and European populations (Gudbjartsson et al., 2015; Mhatre et al., 2017; Boekstegers et al., 2020; Roa et al., 2022). Moreover, a high-fat diet, medication, obesity, and adverse environmental factors contribute to increasing the risk of biliary diseases (Larsson et al., 2017). However, the specific pathogenesis is not yet clear.

There are approximately 100 trillion microorganisms living in the human intestine, which is a unique genome. Intestinal bacteria have been widely studied. The latest released genome map of the human intestinal microbiota has identified 1,952 species of human intestinal bacteria by reconstructing genomes from 11,850 individuals (Almeida et al., 2019). Based on the classification and identification of bacterial properties, 400 bacterial species were determined to belong to 11 phyla, mainly including Firmicutes, Bacteroidetes, Proteobacteria, and Actinobacteria (Eckburg et al., 2005). Intestinal bacteria are closely associated with human health. When they harmoniously coexist with the human body, they coordinate with each other to maintain internal balance and promote human health. The intestinal microbiota plays an important role through the expression of microbiome genes or by indirectly participating in physiological functions (Tang et al., 2019; Shalon et al., 2023). On the contrary, when the balance between intestinal bacteria and the host is disrupted beyond the body’s regulatory capacity, the intestinal mucosal barrier is breached, consequently triggering inflammatory reactions and leading to various metabolic and immune disorders (Heeney et al., 2018; Round and Mazmanian, 2009; Liu et al., 2015a). With the application of 16S ribosomal RNA (rRNA) sequencing and high-throughput sequencing technology, there has been a more in-depth understanding of the intestinal microbiota. The intestinal microbiota is extensively recognized as a contributing factor to metabolic disorders, and biliary system diseases are generally closely related to metabolic disorders. Therefore, the intestinal microbiota could influence the progression of biliary diseases. It has been reported that approximately 80% of patients with common bile duct stones show *coliform* in the bile (Guman et al., 2022). Currently, there is no systematic review on the association of biliary system diseases and intestinal bacteria. In this article, we will systematically extract data and analyze research results on the relationship between intestinal bacteria and biliary system diseases over the past decade. Furthermore, we delve deeper into the effect of potential pathogenic bacteria on biliary diseases.

## 2 Methods

**Figure 1:**
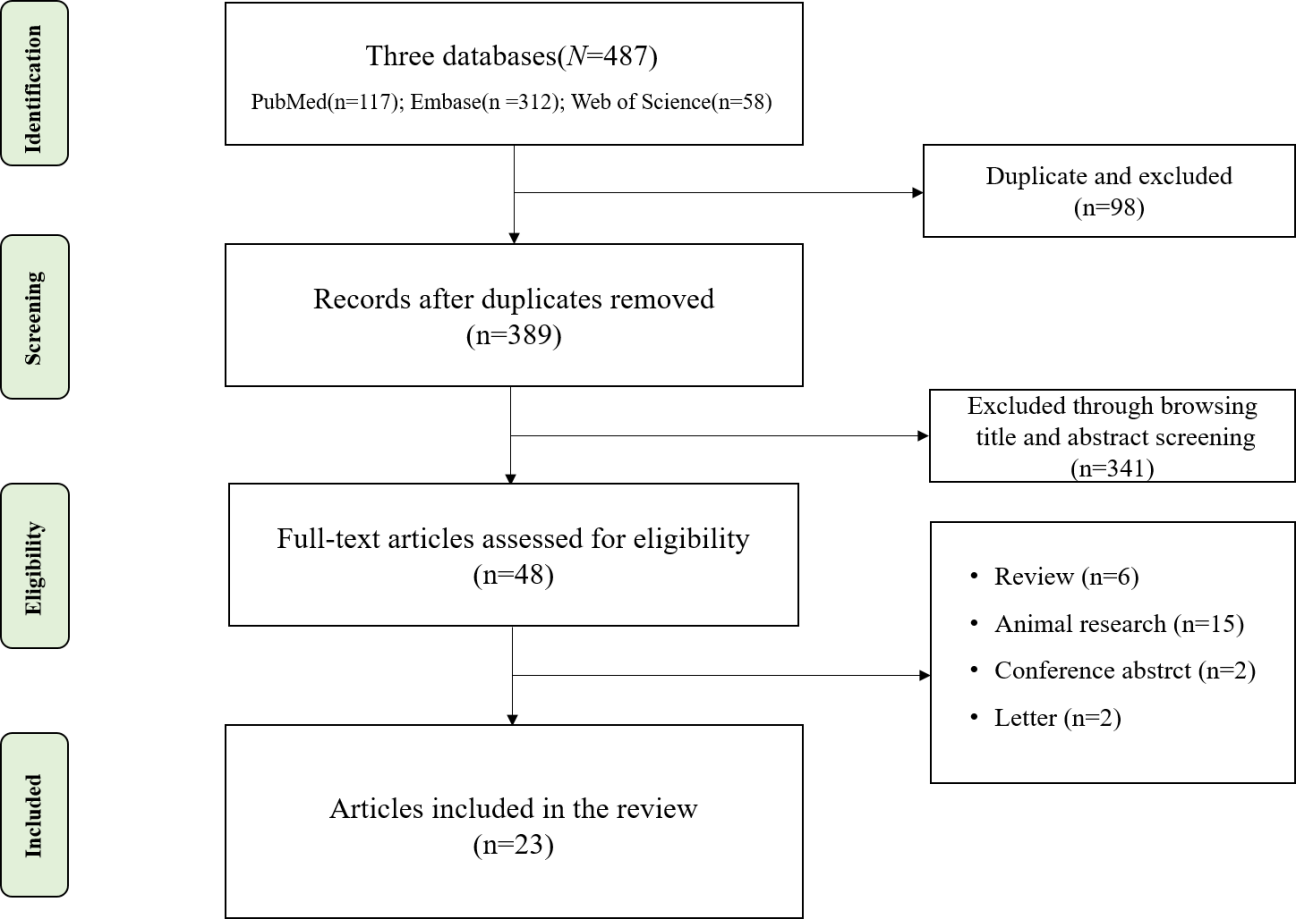
Flowchart of literature screening.

We searched PubMed, Embase, and Web of Science for research studies published up to November 2023. The Preferred Reporting Items for Systematic Reviews and Meta-Analyses (PRISMA) flowchart is shown in **Figure 1**. The inclusion criteria were as follows: 1) case–control or cross-sectional design; 2) patients diagnosed with biliary system diseases and healthy controls (HCs); 3) the study sequenced the intestinal microbiota and reported the relative abundance of the microbiota; and 4) research is reported in English. The exclusion criteria were as follows: case reports, conference abstracts, and animal studies.

## 3 Insights into the biliary system and intestinal bacteria

The biliary system includes the gallbladder and bile ducts, with the latter being categorized into extrahepatic and intrahepatic bile ducts. Extrahepatic bile ducts include the common hepatic duct and the gallbladder duct (Björkström, 2022). The biliary system plays a critical role in the physiology of the host.

1) It participates in the secretion of bile, which is a complex physiological process with the bile components having different secretion pathways. The composition of the bile includes water, inorganic electrolytes, and organic solutes [i.e., bile acids (BAs), cholesterol, phospholipids, and bilirubin]. The production of bile is mainly performed by liver cells, capillaries, and bile duct epithelial cells. Gender, fasting, bile duct pressure, and enterohepatic circulation have an effect on the composition of the bile (Hofmann, 1990; Boyer and Soroka, 2021).
2) It is involved in the transport of bile, with bile production and transport controlled by liver secretion pressure and gallbladder contraction pressure. Once the host fasted, the gallbladder contracts and empties bile while the Oddi sphincter relaxes, resulting in bile being discharged into the duodenum through the gallbladder and common hepatic duct (Pstrpw, 1967). In addition, the gallbladder has concentration, acidification, and secretion functions. The concentration function relies on the reabsorption of water and electrolytes by the gallbladder mucosa (Klaassen and Aleksunes, 2010). The gallbladder bile salt concentration is over 10 times that of liver bile. As the concentration of bile salt increases, the pH of bile decreases. Research has found that the gallbladder mucosa epithelium secretes H^+^ and that bile acidification can inhibit calcified stones (Nakagaki et al., 1984).
3) It participates in the enterohepatic circulation of bile lipid components (e.g., cholesterol, BA, and bilirubin). Cholesterol, BA, and bilirubin are secreted by the liver and are then drained through the biliary tract into the intestine to play physiological roles. After reabsorption in the intestinal wall, they enter the liver through the portal vein. The liver absorbs, binds, and secretes bile to complete the enterohepatic circulation (Hofmann, 1999; Ahmed, 2022). Enterohepatic circulation of the lipid components maintains the host bile concentration stability and avoids biliary system disease occurrence, particularly the formation of GS (Ridlon et al., 2006).

**Figure 2:**
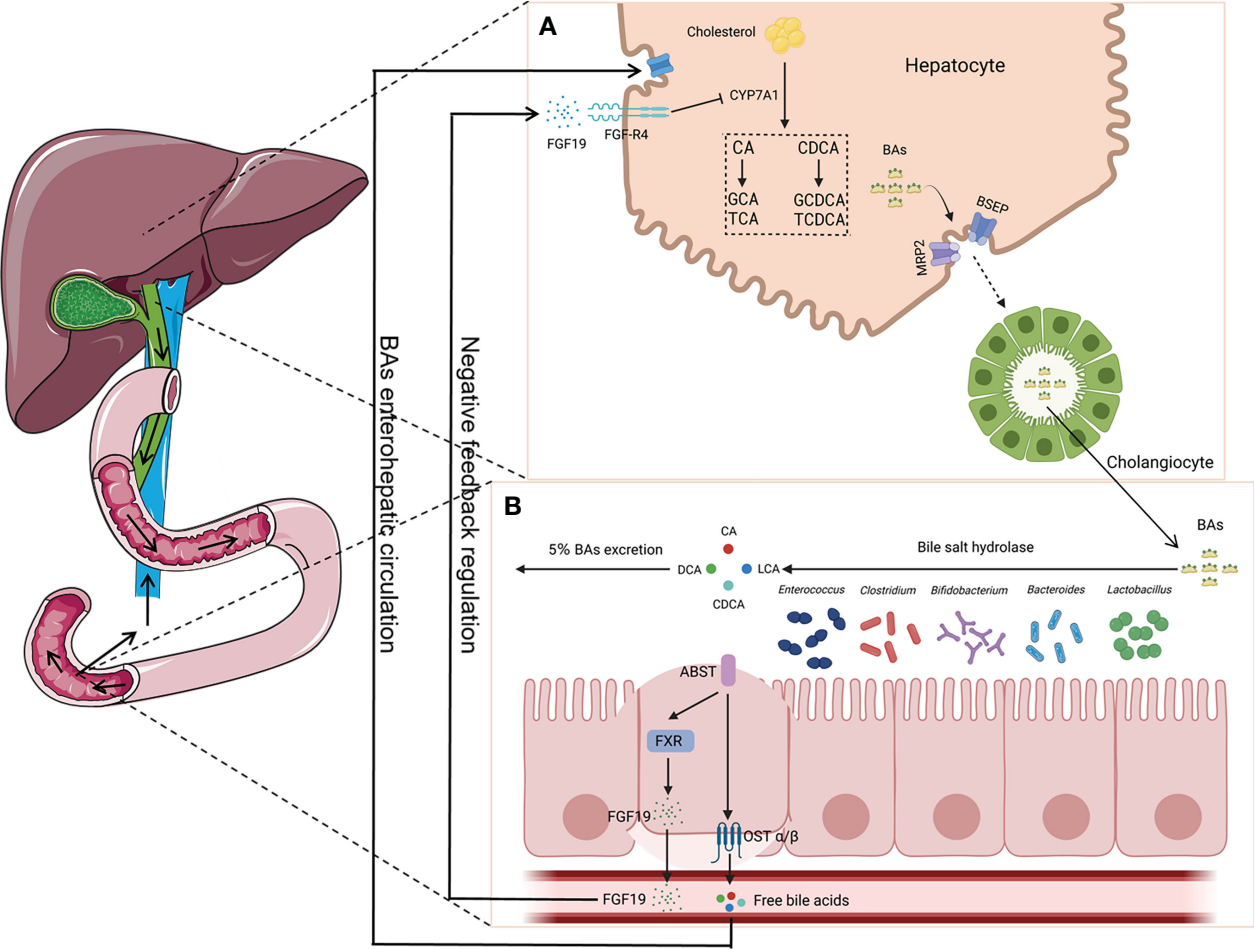
Enterohepatic circulation of bile acids (BAs). **(A)** Primary BAs (CA and CDCA) are synthesized using cholesterol by *CYP7A1* in hepatocytes and then are converted to conjugated BAs (GCA, TCA, GCDCA, and TCDCA) with glycine or taurine, which are secreted into the bile duct by translocators (MRP2 and BSEP). **(B)** BAs are hydrolyzed into CA, CDCA, LCA, and DCA by bacteria (i.e., *Bacteroides*, *Clostridium*, *Bifidobacterium*, *Lactobacillus*, and *Enterococcus*), which can produce bile salt hydrolase (BSH), with most of them being reabsorbed back into the liver. The reabsorption process activates the farnesoid X receptor (FXR) and produces fibroblast growth factor 19 (FGF19), which plays a key role in the maintenance of BA metabolism dynamic balance. CA, cholic acid; CDCA, chenodeoxycholic acid; GCA, glycocholic acid; TCA, taurocholic acid; GCDCA, glycochenodeoxycholic acid; TCDCA, taurochenodeoxycholic acid; LCA, lithocholic acid; DCA, deoxycholic acid; MRP2, multidrug resistance-associated protein 2; BSEP, bile salt export pump.

Presently, the microbiota communities in different parts of the mammalian gastrointestinal tract have been extensively studied. However, the characteristics of the microbiota in the bile duct are unclear. Jiménez et al. employed culture-dependent techniques and analysis based on 16S rRNA genes in the bile and gallbladder mucus of healthy pigs. They found that Proteobacteria, Firmicutes, and Bacteroidetes are the primary phyla in pigs (Jiménez et al., 2014). Researchers have also explored the characteristics of the biliary microbiota using bile samples from healthy dogs and rabbits. It was found that the genus *Enterococcus* was present in healthy dogs (Kook et al., 2010). Moreover, it was observed that Firmicutes, Bacteroidetes, and Proteobacteria were the primary phyla in rabbits (Xing et al., 2019). Molinero’s team reported that Firmicutes, Bacteroidetes, Actinobacteria, and Proteobacteria were present in the bile duct of the population without any liver or gallbladder diseases. Furthermore, compared with patients with GS, the abundance of Propionibacteriaceae was relatively higher in the population without any liver or gallbladder diseases (Molinero et al., 2019). The above-mentioned research studies suggest that there might be microorganisms in the healthy bile duct and that they maintain the balance with the host. Based on existing research findings, intestinal bacteria have an influence on BA metabolism. BAs can promote the emulsification and absorption of fats and activate receptors. Cholesterol in the liver is converted into primary BAs (e.g., cholic acid and chenodeoxycholic acid) by cholesterol 7α-hydroxylase (*CYP7A1*). In addition, it forms conjugated BAs with glycine or taurine in the liver, then secreted into the bile (Russell, 2003). A portion of the conjugated BAs are hydrolyzed into free BAs by bile salt hydrolase (BSH), which is produced by intestinal bacteria. The identified bacteria with BSH included *Bacteroides*, *Clostridium*, *Bifidobacterium*, *Lactobacillus*, and *Enterococcus* (O’Flaherty et al., 2018). Most of the free BAs are reabsorbed back into the liver, while a small portion undergoes dehydroxylation by the intestinal microbiota, forming secondary BAs such as deoxycholic acid and lithocholic acid (Jia et al., 2018). The reabsorption process of free BAs in the intestine activates the farnesoid X receptor (FXR) and promotes the production of fibroblast growth factor 19 (FGF19). After reaching the liver through portal vein circulation, FGF19 interacts with the fibroblast growth factor receptor 4 (FGF-R4) on the liver cell membrane, inhibiting the expression of *CYP7A1*, thereby reducing BA synthesis and maintaining BA metabolism dynamic balance (Sayin et al., 2013). However, opportunistic pathogens exist in the host’s intestine. These are contagious pathogens that typically coexist with humans and can colonize other parts of the body. To the best of our knowledge, *Bacteroides fragilis* is a bile-resistant microorganism and is an opportunistic pathogen. When the body’s immune system is weakened or during microbial disorders, *B. fragilis* can migrate from the intestine to the bile duct and induce infection (**Figure 2**) (Chung et al., 2018; Yang et al., 2022).

## 4 Gallstones and intestinal bacteria

There are many types of GS, but there is currently no classification standard. Many scholars have advocated classifying stones according to their chemical composition, generally divided into cholesterol stones and bile pigment stones. Cholesterol stones account for approximately 80% of GS in the European and American populations. Existing theories suggest that the formation of cholesterol stones is mainly caused by the supersaturation of cholesterol in the bile and that the cholesterol content is more than 60% (Van Erpecum, 2011; Lammert et al., 2016). The cholesterol content in bile pigment stones is lower than 40%–45%, which is mainly composed of “bilirubin calcium,” a product of calcium combined with bilirubin. Analysis of the primary components has great value in exploring the etiology of GS. The biliary tract downstream is connected to the intestine, which is a complex internal environment. Ding et al. collected fecal samples from 42 patients with GS and 20 HCs using the metagenomic next-generation sequencing method and found that, compared with that in the HC group, the phylum Bacteroidetes and the genera *Bacteroides*, *Prevotella*, *Barnesiella*, *Odoribactor*, and *Tannerella* had relatively higher abundance in GS (Ding et al., 2023). Song et al. also observed differences in the bacterial structure between patients with GS and HCs. The abundance of bacteria in patients with GS significantly increased, while the diversity of bacteria was decreased. At the phylum level, compared to HCs, the relative abundance of Firmicutes was increased, while Bacteroidetes and Proteobacteria were decreased in GS. At the genus level, the abundance of *Sutterella*, *GCA-900066755*, *Butyricicoccus*, *unclassified_O_Lactobacillales*, and *Lachnospiraceae_ND3007_group* was significantly reduced in GS. However, the abundance of *Megamonas*, *Comamonas*, *Coprobacillus*, *Adlercreutzia*, *unclassified_P_Firmicutes*, *Morganella*, and *CHKCI002 Tyzzerella_4* was significantly increased in GS (Song et al., 2022). Hu et al. reported that the abundance of Bacteroidetes was also decreased in GS, while the relative abundance of Desulfovibrionales was increased (Hu et al., 2022). Wang’s team used a 16S rRNA gene sequencing method to analyze fecal sample DNAs between GS patients and HCs. The abundance of Firmicutes was significantly reduced in patients with GS, with the Firmicutes/Bacteroidetes ratio also reduced. On the other hand, the abundance of *Rhododocus*, *Treponema_2*, *Wolbachia*, *Ochrobactrum*, *Rubus_Hybrid_Cultiva*, *Ruminicostridium_9*, and *Eisenbergiella* was increased, but that of *Faecalibacterium* was decreased (Wang et al., 2020). Wu et al. also found that the abundance of *Faecalibacterium* was decreased in patients with GS (Wu et al., 2013). The above findings indicate that patients with GS have a phenomenon of intestinal bacteria disorder. For specific data, refer to the **Supplementary material**.

**Figure 3:**
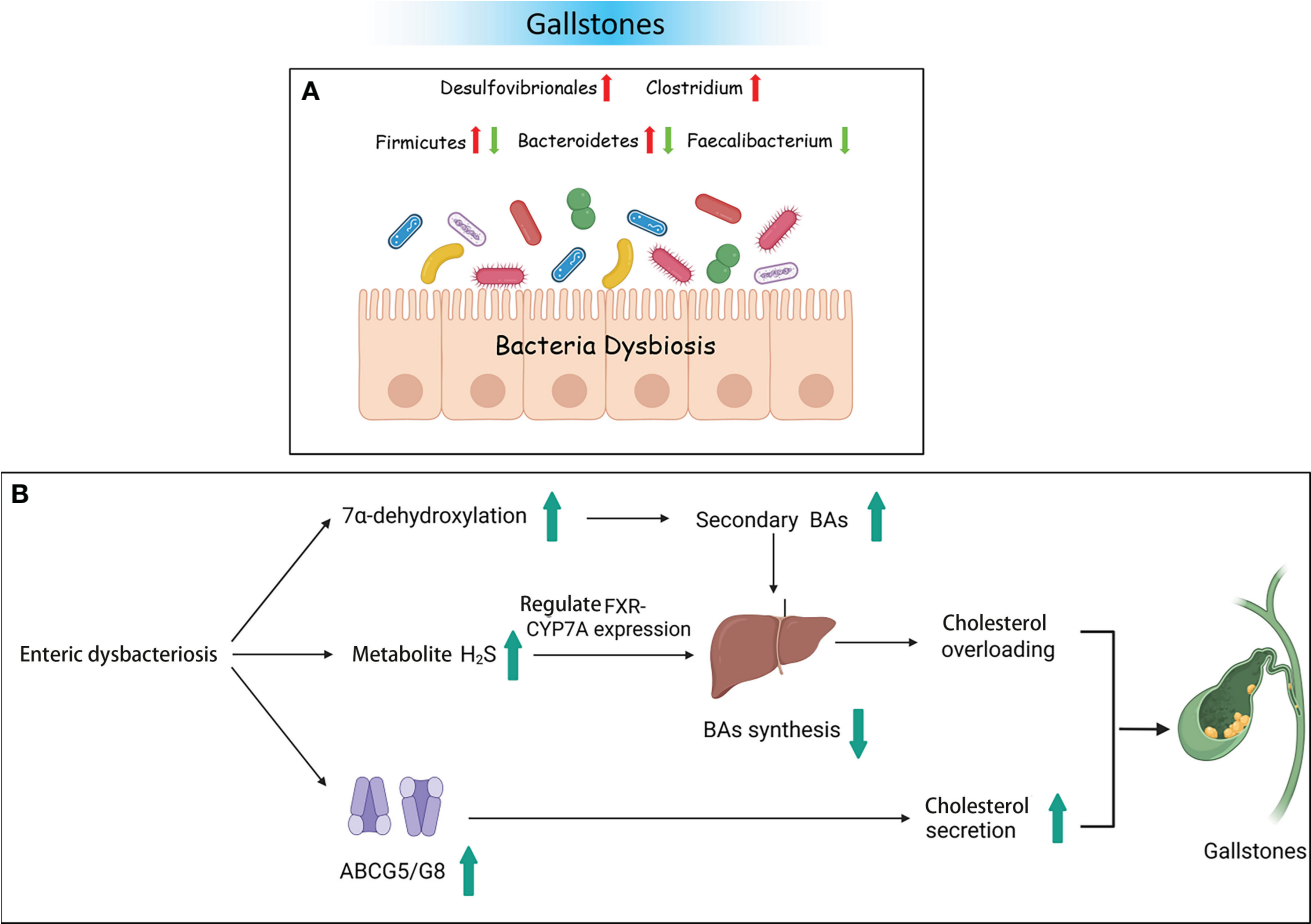
Gallstones and the intestinal microbiota. **(A)** Bacterial dysbiosis in patients with gallstones. **(B)** Some possible mechanisms of gallstones resulting from dysbacteriosis.

Moreover, numerous researchers have validated bacterial changes in GS using animal models and explored the potential mechanisms of pathogenic bacteria. A study on an animal model given a lithogenic diet observed that the bacterial richness and *α* diversity were decreased. The abundance of Firmicutes and the ratio of Firmicutes/Bacteroidetes were significantly decreased. At the genus level, the relative abundance of *Akkermansia*, *Clostridium_XlVa*, and *Clostridium_XVIII* was significantly increased. However, the abundance of *Acetivibrio*, *Ruminococcus*, and *Lactobacillus* was significantly decreased (Wang et al., 2017). Research also showed that free BAs are converted into secondary BAs by bacteria with 7α-dehydroxylase activity. It is known that the genera *Clostridium*, *Trichinellidae*, and *Streptococcus* possess 7α-dehydroxylase activity (Ridlon et al., 2016). Therefore, enrichment of *Clostridium_XlVa* and *Clostridium_XVIII* could lead to increasing the level of 7α-dehydroxylase, thereby increasing the level of secondary BAs. Finally, there is excessive inhibition of BA synthesis. Moreover, a decrease in the number of *Lactobacillus* was not conducive to reducing the levels of cholesterol (Cui et al., 2022). Another mouse experiment reported opposite results. Compared to the normal control group, the abundance of the phylum Firmicutes, the proportion of Firmicutes/Bacteroidetes, and the genus *Desulfovibrio* were significantly increased in a lithogenic mouse model. The relative abundance of *Bacteroidetes* and *Prevotella* was decreased (Li et al., 2023). Hu et al. found that Desulfovibrionales was enriched in patients with GS. When transplanted into GS-resistant mice intestines, it then induced the formation of GS. The study also found that enrichment with Desulfovibrionales contributed to improved levels of secondary BAs in the cecum and increased the hydrophobicity of BAs, which caused an increase in the reabsorption of cholesterol in the intestine. On the other hand, mice carrying Desulfovibrionales induced liver expression of the cholesterol transporter protein Abcg5/g8, promoting cholesterol secretion. Not only do bacteria play a crucial role in the development of GS, but bacterial metabolites have also been proven to be involved in GS. The metabolite H_2_S of Desulfovibrionales could induce liver FXR and inhibit the expression of *CYP7A1*, leading to BA synthesis increase and cholesterol supersaturation, thus facilitating the formation of stones (**Figure 3**) (Hu et al., 2022).

## 5 Non-calculous cholangitis and intestinal microbiota

The risk factors for cholangitis include GS and bacterial, viral, and parasitic infections. In this article, we focused on non-calculous cholangitis [i.e., cholecystitis, primary sclerosing cholangitis (PSC), and primary biliary cholangitis (PBC)]. Acute cholecystitis (AC) leads to inflammatory lesions, with clinical manifestations including fever, right upper abdominal pain, and increased peripheral white blood cell count. Research has observed that bacterial infection activates the coagulation protein factor XII to produce arteritis, causing vascular damage. Factor XII produces bradykinin, which promotes the occurrence of AC (Poddighe and Sazonov, 2018; Hanabata et al., 2022). A study found that *Escherichia coli* accounted for approximately 73.7% of the bacterial cultures in the bile of patients with AC (Kujiraoka et al., 2017). Another study found a positive correlation of Enterobacteriaceae with the intestine and bile of patients with AC. Bile endotoxins were correlated with Enterobacteriaceae, particularly with the abundance of *E. coli*. The study also found that controlling the number of Enterobacteriaceae can reduce the risk of gallbladder infection (Liu et al., 2015b). Chronic cholecystitis is a long-term inflammation that contributes to fibrous thickening and mucosal hyperplasia. Bacterial infections and abnormal cholesterol metabolism can also contribute to the occurrence of chronic cholecystitis. Studies have shown that 20%–30% of patients with chronic cholecystitis are bile bacterial culture-positive. Researchers have proposed that bacterial infection may be transmitted through the portal vein system or the intestinal lymphatic system (Treem et al., 1989).

**Figure 4:**
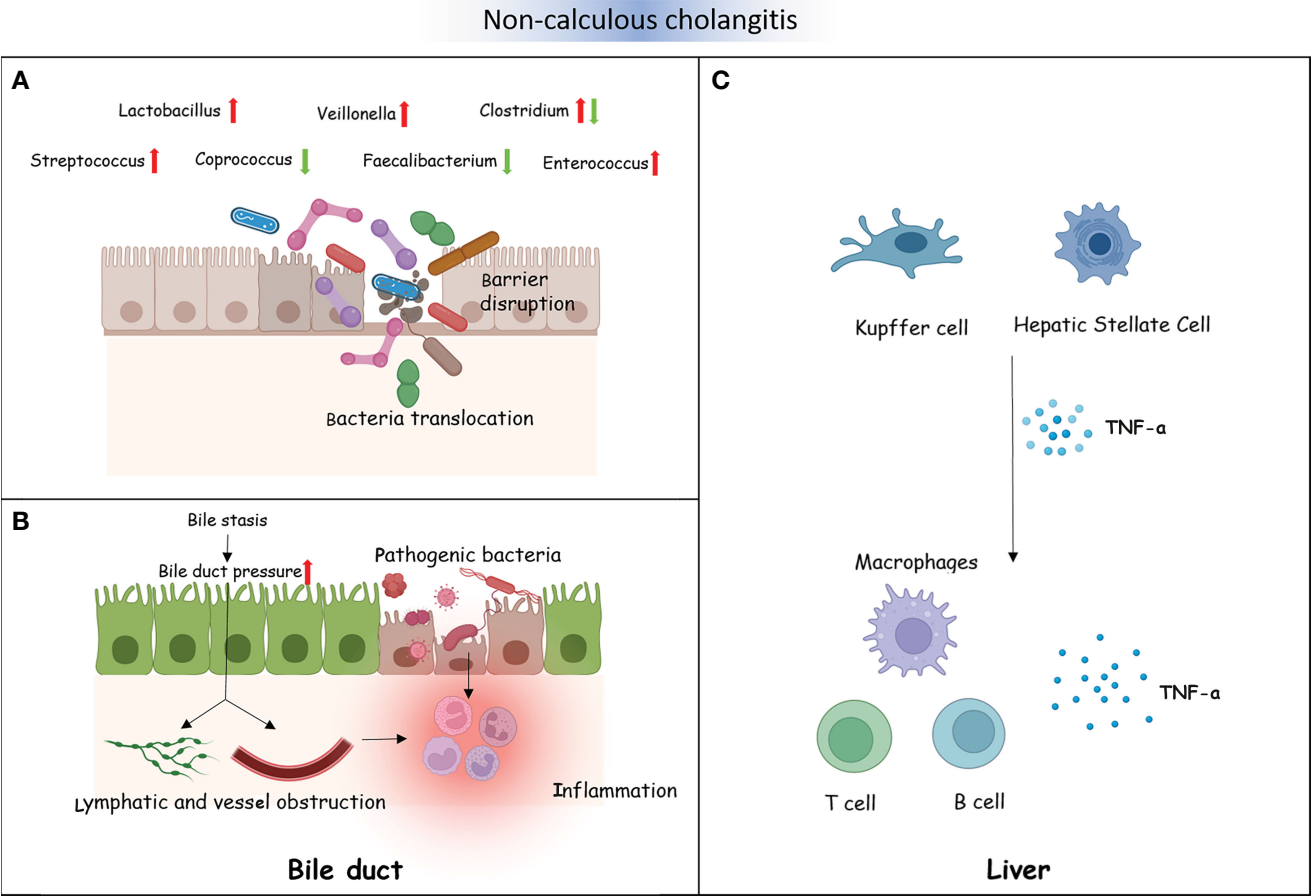
Non-calculous cholangitis and the intestinal microbiota. **(A)** The intestinal barrier is disrupted by some gut microbiota in patients with non-calculous cholangitis. A number of intestinal microorganisms or their metabolites could translocate through the damaged intestinal epithelial barrier. **(B)** Bacterial dysbiosis influences bile acid (BA) metabolism, which causes changes in the bile composition, resulting in bile stasis and bile duct pressure increase. This will disrupt the lymphatic and vascular flow and lead to inflammation **(A)**. Several translocated pathogenic bacteria and their metabolites destroy the cholangiocytes, resulting in inflammatory reactions **(B)**. **(C)** Translocated pathogenic bacteria and their metabolites activate the liver immune cells, including Kupffer cells and hepatic stellate cells, excessively producing pro-inflammatory cytokines and chemokines such as TNF-α. This could recruit immune cells, promoting chronic inflammation of the biliary tract and portal fibrosis, ultimately leading to primary sclerosing cholangitis (PSC).

PSC and PBC are both chronic cholestatic liver diseases, and there is currently no precise treatment. However, there is a difference between PSC and PBC. PSC mainly involves the bold ducts inside and outside the liver. The relationship between PSC and the intestine has a history of over 50 years. Approximately 60%–80% of the Nordic PSC population suffer from inflammatory bowel disease (IBD) (Karlsen, 2016). PBC, on the other hand, mainly affects the small bile ducts in the liver and is not related to IBD. Further research found that, compared to HCs, the diversity of bacteria was decreased in individuals with PSC (Kummen et al., 2017; Liu et al., 2022). Moreover, patients with PSC have fewer bacterial genera (Kummen et al., 2021). An experiment on a PSC mouse model showed that the reduction of bacteria weakened the negative feedback regulation of BAs and then enhanced the accumulation of BAs in the liver. The decrease in bacteria also altered the composition of BAs (Schneider et al., 2021; Awoniyi et al., 2023). In addition, studies have shown that intestinal microbiota dysfunction plays an important role in the progression of PSC (Jin et al., 2022). For example, in PSC mouse models, researchers observed that intestinal microbiota dysfunction through *NLRP3* promoted liver disease progression (Liao et al., 2019). In order to identify potential pathogenic bacteria, we summarized studies on the bacteria between PSC and HC groups (**Supplementary Material**). At the phylum level, two studies from different countries found that the abundance of Firmicutes was significantly decreased in PSC. The study by Sabino et al. showed a significant increase in the abundance of Bacteroidetes in PSC (Sabino et al., 2016). The study by Hole et al. showed a significant increase in the abundance of Proteobacteria in PSC (Hole et al., 2023). At the genus level, although the study populations were from different countries, multiple studies have reported a significant decrease in the abundance of *Faecalibacterium*, *Eubacterium*, and *Coprococcus* in PSC (Karlsen, 2016; Rühlemann et al., 2019; Lapidot et al., 2021). These three genera are important producers of butyric acid, which has anti-inflammatory effects and protects the digestive system from intestinal pathogens. In contrast, the abundance of *Veillonella*, *Clostridium*, *Lactobacillus*, *Streptococcus*, *Enterococcus*, and *Blautia* significantly increased in PSC (Sabino et al., 2016; Torres et al., 2016; Iwasawa et al., 2017; Kummen et al., 2017; Rühlemann et al., 2019; Kummen et al., 2021; Lapidot et al., 2021; Liu et al., 2022; Hole et al., 2023). *Veillonella* could enter the liver through intestinal lymphocytes. It has been proven to be associated with inflammation and progressive fibrosis (Molyneaux et al., 2014). *Clostridium* and *Lactobacillus* could improve the level of BSH and induce the increase of primary BAs. *Streptococcus* is a common bacterium that causes human purulent inflammation and toxic and hypersensitive diseases. *Enterococcus* secretes metalloproteinases to break down epithelial cadherin, thereby disrupting the intestinal barrier. Intestinal microorganisms or their products undergo translocation through the damaged intestinal epithelial barrier, thereby inducing the activation of immune cells in the liver (Gaca and Lemos, 2019). The activation of Kupffer and hepatic stellate cells results in the excessive production of pro-inflammatory cytokines and chemokines (such as tumor necrosis factor). This inflammatory response can cause chronic inflammation of the biliary tract and portal fibrosis, ultimately leading to PSC. Silva et al. found that an increase in *Enterococcus* was positively associated with serum alkaline phosphatase (ALP) levels and bile duct obstruction (Vieira-Silva et al., 2019). Due to the inherent resistance of *Enterococcus*, treatment is difficult. The genus *Blautia* participates in multiple sclerosis through immune regulation and pro-inflammatory pathways. It has an impact on the activation of antigen-presenting B and T cells and the upregulation of complement activation-related genes. Similar to the intestinal bacteria of patients with PSC, the diversity of the intestinal microbiota of patients with PBC was also significantly reduced compared to HCs (Zhou et al., 2023). A comparative study between 76 patients with PBC and 23 HCs observed that *Clostridium* was decreased in PBC, while *Lactobacillus*, *Streptococcus*, and *Enterococcus* were increased (Furukawa et al., 2020). Another study reported *Pseudomonas* to be significantly increased in PBC (Kitahata et al., 2021). Tang’s research team also observed *Pseudomonas* as increased in patients with PBC by using the same sequencing method. They found that the abundance of *Veillonella*, *Clostridium*, *Lactobacillus*, *Haemophilus*, *Streptococcus*, and *Klebsiella* was increased, while that of *Sutterella*, *Faecalibacterium*, and *Oscillospira* was decreased. The research results of Zhou et al. were consistent with those of Tang et al., that *Faecalibacterium* was significantly reduced in PBC. However, *Acidimicrobium* and *Serratia* significantly increased in PBC (Zhou et al., 2023). Therefore, the same potential pathogenic bacterial genera (i.e., *Faecalibacterium*, *Veillonella*, *Clostridium*, and *Lactobacillus*) were found to be common between PSC and PBC. The interaction between bacterial imbalance and BAs plays a promoting role in the pathogenesis of PBC. Research has shown a negative correlation between *Veillonella* and secondary BAs in PBC. In contrast, *Faecalibacterium* is positively correlated with secondary BAs in HCs (**Figure 4**) (Chen et al., 2020).

In summary, the impact of intestinal pathogenic bacteria on the development of non-calculous cholangitis was explored in three aspects, as follows:

1) Pathogenic bacteria that directly ascend to the extrahepatic bile duct through the intestine. In healthy hosts, the normal drainage of bile makes it difficult for pathogenic bacteria to retrograde into the common bile duct. However, when bile stasis occurs, it is difficult to eliminate bacteria, which constitutes the basic condition for biliary tract infection.
2) The hypothesis of “intestinal leakage” refers to the excessive production of pro-inflammatory cytokines and chemokines in the liver by pathogenic bacteria and their products (through the damaged intestinal barrier). This may lead to cholangitis. However, bacterial translocation to the liver can also promote the occurrence of bile duct inflammation (Ma et al., 2018; Xia et al., 2023).
3) Due to the imbalance of bacteria involved in BA metabolism in a patient’s intestine, the intestinal hepatic circulation of BAs is affected, causing changes in the bile composition. The accumulated bile blocks the bile duct, causing bile stasis and increasing the pressure inside the bile duct. This will cause the bile duct wall to expand, ultimately disrupting the lymphatic and vascular flow and producing inflammatory reactions.

## 6 Biliary tract cancer and intestinal bacteria

**Figure 5:**
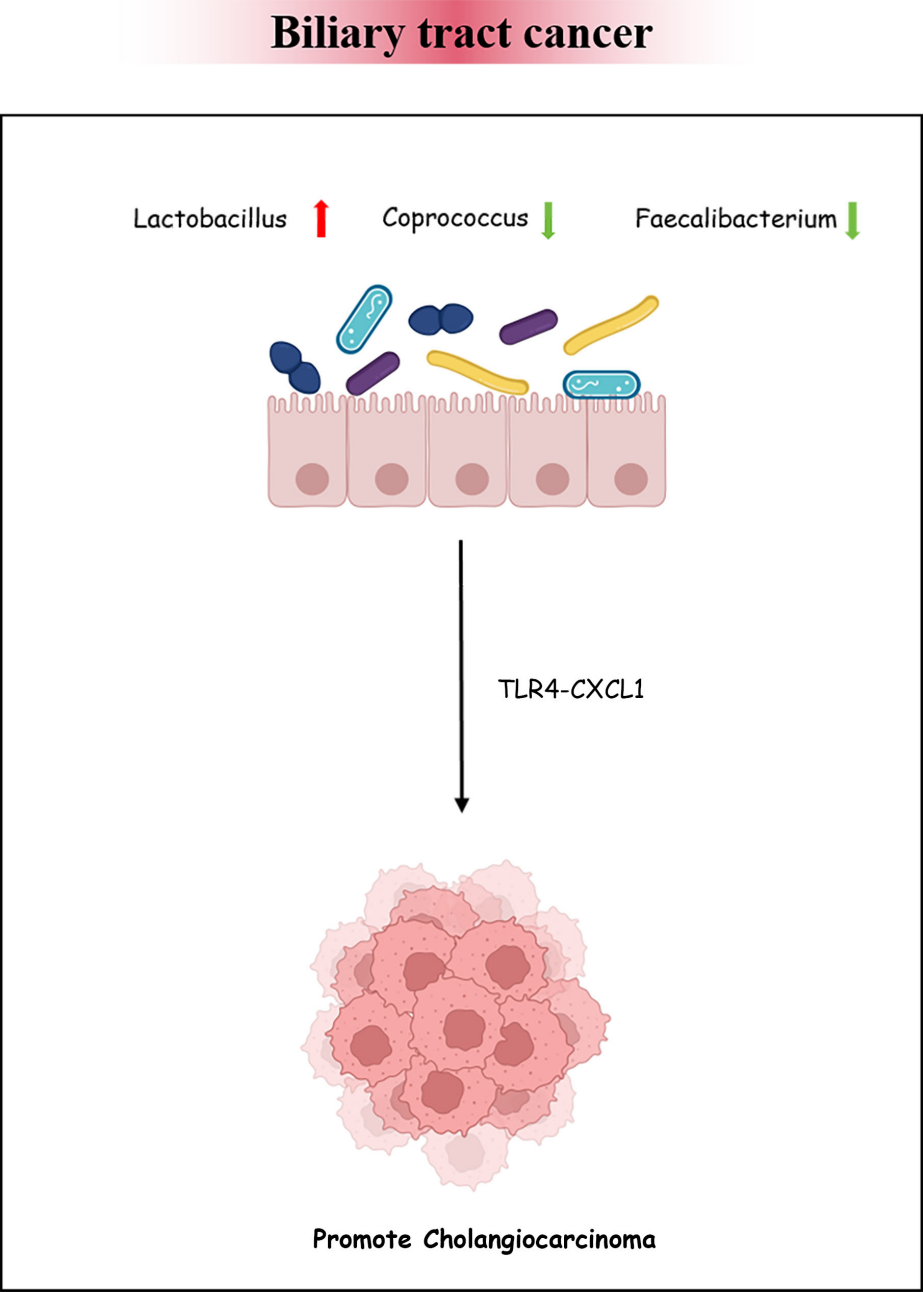
Biliary tract cancer and the intestinal microbiota. Bacterial dysbiosis could promote cholangiocarcinoma formation by regulating the *TLR4*–*CXCL1* axis.

BTC has an invisible onset and lacks early effective diagnostic measures. Once identified, the progression of the disease would have been at an aggressive stage and have a poor prognosis. More and more evidence has suggested that intestinal bacteria have an impact on the occurrence and development of various cancers (Saud Hussein et al., 2021). There is insufficient theoretical basis for the association between BTC and intestinal bacteria. We retrieved three articles exploring the characteristics of bacteria in BTC. The enriched genera in BTC mainly included *Bacteroides*, *Lactobacillus*, *Gammaproteobacteria*, *Actinomyces*, *Alloscardovia, Muribaculum*, and *Alistipes.* However, the abundance of *Coprococcus*, *Clostridium*, *Faecalibacterium*, and *Ruminococcus_1* was decreased (Jia et al., 2020; Zhang et al., 2021b; Ito et al., 2022). The above findings reveal that the intestinal bacteria in patients with BTC are disrupted. Animal experiments have confirmed that the intestinal barrier function is reduced in a PSC mouse model, causing bacteria and lipopolysaccharides to appear in the liver. The expression of *CXCL1* is induced through Toll-like receptor 4-dependent mechanisms in liver cells, and the accumulation of *CXCR2*+ polymorphonuclear myeloid-derived inhibitory cells promotes cancer (Zhang et al., 2021a). The research analysis by Song et al. showed that, during the progression of chronic cholecystitis to BTC, the dominant bacteria in the gallbladder were *Peptostreptococcus stomatis*, *Fusobacterium mortiferum*, *Acinetobacter junii*, and *Enterococcus faecium*. This hints that pathogenic bacteria may persist and endanger host health (**Figure 5**) (Song et al., 2020; Chagani and Kwong, 2021).

## 7 Biomarkers and therapeutic prospects

We systematically analyzed the potential pathogenic bacteria in biliary system diseases, as shown in **Table 1**. It was found that the abundance of *Faecalibacterium*, *Eubacterium*, and *Coprococcus* was generally reduced. Particularly, *Faecalibacterium* was decreased in GS, PSC, PBC, and BTC, while *Veillonella*, *Lactobacillus*, *Streptococcus*, and *Enterococcus* were significantly increased. *Lactobacillus* showed an increase in PSC, PBC, and BTC. Interestingly, we found that the relative abundance of *Clostridium* was generally reduced in GS, PBC, and BTC. However, it was generally increased in PSC. These findings imply that intestinal bacterial changes could provide a basis for early diagnosis. For biliary tract diseases, symptomatic treatments are selected clinically. However, they cannot be cured in a timely manner. With in-depth research on intestinal bacteria, many scholars have proposed reconstructing the homeostasis of the intestinal microbiota, which has profound significance for disease prevention and treatment. Allegretti et al. performed fecal transplantation for the first time in 10 patients with PSC, which increased the diversity of the intestinal microbiota. Importantly, *Odoribacter*, *Alistipes*, and *Erysipelotrichaceae incertae sedis* were found to be correlated with decreased ALP levels in patients post-fecal microbiota transplantation (FMT) (Allegretti et al., 2019). At present, studies with larger sample sizes are needed to further understand the association between intestinal bacteria and biliary diseases. Animal experiments should be improved to verify the mechanisms of potential pathogenic bacteria and the effectiveness of targeted microbial therapy in order to improve the level of early diagnosis of diseases and provide more treatment directions for biliary diseases in the future.

**Table 1:** Bacterial genera with significant changes in biliary diseases compared with healthy controls (*N* ≥ 3).

Disease	First author	Region	Year of publication	Design	Samples	Cases	Healthy control	Method	*Bacteroides*	*Faecalibacterium*	*Eubacterium*	*Veillonella*	*Clostridium*	*Lactobacillus*	*Streptococcus*	*Coprococcus*	*Enterococcus*
GS	Wu (Wu et al., 2013)	China	2013	Case-control	Feces	29(49.2 ± 14.3)	38(40.7 ± 14.5)	16S rRNA sequencing		↓							
GS	Wang (Wang et al., 2020)	China	2020	Case-control	Feces	30(42.6 ± 12.5)	30(40.5 ± 11.5 )	16S rRNA sequencing		↓	↓						
GS	Ding (Ding et al., 2023)	China	2023	Case-control	Feces	42(49.69 ± 7.37)	20(46.10 ± 6.32)	Metagenomic Sequencing	↑								
AC	Liu (Liu et al., 2015b)	China	2015	Case-control	Feces	15(38-79)	13(38-79)	16S rRNA sequencing					↓			↓	
PSC	Rossen (Rossen et al., 2015)	Netherlands	2015	Case-control	Mucosal	12(29.5)	9(65)	16S rRNA sequencing					↓				
PSC	Sabino (Sabino et al., 2016)	Belgium	2016	Case-control	Feces	18(49)	66(51.5)	16S rRNA sequencing						↑	↑		↑
PSC	Kummen (Kummen et al., 2017)	Norway	2017	Case-control	Feces	85(49)	263(46.5)	16S rRNA sequencing				↑	↑				
PSC	Iwasawa (Iwasawa et al., 2017)	Japan	2017	Case-control	Feces	27(12)	23(12)	16S rRNA sequencing				↑			↑		↑
PSC	Rühlemann (Rühlemann et al., 2019)	Germany	2019	Case-control	Feces	62(51)	133(49)	16S rRNA sequencing		↓		↑		↑	↑	↓	↑
PSC	Kummen (Kummen et al., 2021)	Norway	2021	Case–control	Feces	136 (–)	158 (–)	Metagenomic sequencing			↓		↑				
PSC	Lapidot (Lapidot et al., 2021)	Israel	2021	Case–control	Feces	17 (52.24)	30 (58.6)	16S rRNA sequencing		↓			↑	↑			↑
PSC	Liu (Liu et al., 2022)	China	2022	Case–control	Feces	37 (–)	64 (–)	16S rRNA sequencing		↓	↓	↑		↑		↓	
PSC	Hole (Hole et al., 2023)	Norway	2023	Case–control	Mucosal	84 (40)	40 (62)	16S rRNA sequencing				↑					
PBC	Furukawa (Furukawa et al., 2020)	Japan	2020	Case–control	Feces	76 (66.0 ± 8.3)	23 (60.5 ± 8.1)	16S rRNA sequencing					↓	↑	↑		↑
PBC	Tang (Tang et al., 2018)	China	2022	Case–control	Feces	60 (51.5)	80 (48)	16S rRNA sequencing	↓	↓		↑	↑	↑	↑		
PBC	Zhou (Zhou et al., 2023)	China	2023	Case–control	Feces	25 (55)	25 (57)	16S rRNA sequencing		↓			↓			↓	
BTC	Jia (Jia et al., 2020)	China	2020	Case–control	Feces	28 (55.32 ± 8.76)	12 (45.32 ± 7.11)	16S rRNA sequencing						↑			
BTC	Zhang (Zhang et al., 2021b)	China	2021	Case–control	Feces	53 (67.1)	40 (53.6)	16S rRNA sequencing	↑					↑			
BTC	Ito (Ito et al., 2022)	Japan	2022	Case–control	Feces	30 (75.5)	10 (63.5)	16S rRNA sequencing		↓			↓			↓	

↑The relative abundance of bacteria was increased. ↓The relative abundance of bacteria was decreased.

N, number of articles reported on biliary system diseases; GS, gallstones; AC, acute cholecystitis; PSC, primary sclerosing cholangitis; PBC, primary biliary cholangitis; BTC, biliary tract cancer.

(–), ave. age was not provided.

## Data availability statement

The original contributions presented in the study are included in the article/**Supplementary Material**. Further inquiries can be directed to the corresponding authors.

## Author contributions

HW: Writing – original draft. JG: Writing – original draft. JC: Data curation, Writing – review & editing. WZ: Data curation, Writing – review & editing. YS: Project administration, Writing – review & editing. DS: Project administration, Writing – review & editing.
